# Alternative splicing and cancer: a systematic review

**DOI:** 10.1038/s41392-021-00486-7

**Published:** 2021-02-24

**Authors:** Yuanjiao Zhang, Jinjun Qian, Chunyan Gu, Ye Yang

**Affiliations:** 1 The Third Affiliated Hospital of Nanjing University of Chinese Medicine, Nanjing, China; 2 School of Medicine & Holistic Integrative Medicine, Nanjing University of Chinese Medicine, Nanjing, China

**Keywords:** RNA splicing, Non-coding RNAs, Molecular medicine, Drug development

## Abstract

The abnormal regulation of alternative splicing is usually accompanied by the occurrence and development of tumors, which would produce multiple different isoforms and diversify protein expression. The aim of the present study was to conduct a systematic review in order to describe the regulatory mechanisms of alternative splicing, as well as its functions in tumor cells, from proliferation and apoptosis to invasion and metastasis, and from angiogenesis to metabolism. The abnormal splicing events contributed to tumor progression as oncogenic drivers and/or bystander factors. The alterations in splicing factors detected in tumors and other mis-splicing events (i.e., long non-coding and circular RNAs) in tumorigenesis were also included. The findings of recent therapeutic approaches targeting splicing catalysis and splicing regulatory proteins to modulate pathogenically spliced events (including tumor-specific neo-antigens for cancer immunotherapy) were introduced. The emerging RNA-based strategies for the treatment of cancer with abnormally alternative splicing isoforms were also discussed. However, further studies are still required to address the association between alternative splicing and cancer in more detail.

## Introduction

Since the first discovery of eukaryotic “split” genes in 1977 harboring the intervening sequences, it has been known that these intron sequences could be removed by the spliceosome complex.^1,2^ Increasing evidence has shown that ~94% of human genes have intronic regions during the pre-mRNA processes, and most eukaryotic genes undergo alternative splicing in a tempo-spatial-dependent manner,^3^ which is regulated by multiple RNA-binding proteins (RBPs) and depends on *cis-*acting elements and *trans*-acting factors. Different mature messenger RNA (mRNAs) with different functions could, therefore, be synthesized from a single gene through alternative splicing, which increases the complexity of mRNA for the diversity of proteins.^4,5^

Recently, it was reported that splicing may be closely associated with the occurrence of tumors, and that abnormal changes in alternative splicing could affect tumor progression.^6^ It could also disrupt the protein interaction pathways in tumor development.^7,8^

In the present review, the complexity of the splicing network, including *cis*-elements, spliceosome assembly and a plethora of trans-elements with antagonistic functions that define RNA maturation were first explained. Next, the function of alternative splicing as an oncogenic driver and/or passenger during tumor cell progression adaptive responses to metabolic stress and neo-angiogenesis was discussed, with emphasis on hematological malignancy. Finally, recent advances in therapeutics targeting splicing catalysis and splicing regulatory proteins were summarized, and emerging novel technologies were discussed using RNA-based therapies to modulate pathogenically spliced isoforms.

## Regulatory splicing components and alternative splicing

The process of removing introns from pre-mRNAs and connecting the remaining exons to each other to produce mature mRNA is called splicing, while the different combination of exons in the mRNA producing diversified mature mRNA is called alternative splicing.^9^ RNA splicing can be accomplished inside the nucleus by an enzymatic machine termed spliceosome. Spliceosome mainly recognizes the junction of introns and exons, and generally follows the “GU-AG” rule to distinguish introns from exonic sequences by four canonical consensus sequences: (i) The 5′ splice-site (SS; characterized by a GU dinucleotide at the 5′ end of the intron), (ii) the 3′ SS (containing AG at the 3′ end of the intron), (iii) the branch point sequence (BPS; located upstream of the 3′ SS), and (iv) the polypyrimidine tract (located between the BPS and the 3′ SS).^10^ Alternative splicing depends on many *trans*-acting factors as well as certain *cis-*acting elements.^11^

### Core spliceosome machinery

**Fig. 1:**
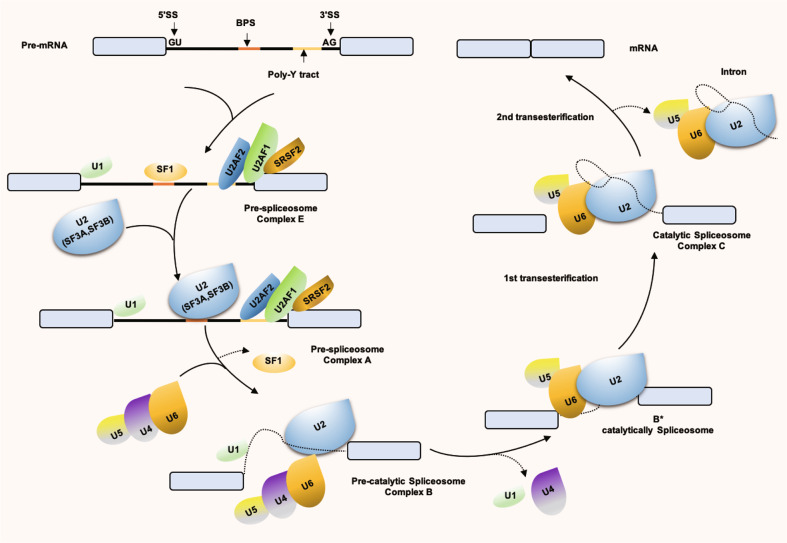
Spliceosome assembly. U1 snRNP recognizes 5′ SS and binds through base-pairing, SF1 binds to BPS, U2AF2 binds to polypyrimidine tract and U2AF1 binds to 3′ SS, forming Complex E. Next, U2 snRNP, with the assistance of U2AF, replaces SF1 with the BPS through base-pairing to form Complex A. Next, U5/U4/U6 is recruited and results in the rearrangement of Complex A. Among them, U4 and U6 snRNP are combined through complementary pairing of their RNA components, while U5 snRNP is loosely bound through protein interaction, at which time a Complex B is formed. Through a series of conformational changes, U1 snRNP leaves, U6 snRNP binds to 5′ SS, and at the same time, U4 snRNP leaves so that U6 snRNP and U2 snRNP pair through snRNA. After this rearrangement process, Pre-catalytic Spliceosome Complex B is formed, followed by two transesterification reactions. The first transesterification reaction generates Complex C. The rearrangements occur in Complex C, promoting second transesterification, resulting in a post-spliceosomal complex. As a result, exons are interconnected to form mature mRNA, introns are degraded and snRNPs are recycled. SF1 splicing factor 1, BPS branch point sequence, SS splice-site, snRNPs small nuclear RNPs

Alternative splicing is a regulated process, mainly executed by the spliceosome machinery and affected by the activity of splicing regulators, such as serine/arginine-rich (SR) proteins or heterogeneous nuclear ribonucleoproteins (hnRNPs), which are the most important mediators of SS recognition.^12^ The spliceosome is composed of 5 small nuclear RNPs (snRNPs; U1, U2, U4, U5, and U6 snRNPs), each of which contains its own snRNA complexed to a group of 300 associated proteins.^13,14^ Thanks to the recent structural studies using cryo-electron microscopy, an unprecedented high-resolution view of each step was obtained during spliceosome body assembly.^15–18^ These snRNPs bind to pre-mRNA, as shown in Fig. 1, U1 snRNP binds to 5′ SS, and U2 (including SF3A, SF3B) binds to 3′ SS and polypyrimidine sequences. Next, U5, U4/U6 were recruited, and a catalytically active complex was formed by rearrangement between snRNPs to complete intron excision and exon linkage.^19,20^

The SR proteins^21^ and hnRNP family^12^ are two essential auxiliary factors in enhancing or repressing splice-site usage by the recognition of specific *cis-*acting RNA elements. In general, SR proteins play a positive role in splicing regulation and preferentially bind to exonic splicing enhancer (ESE) and intronic splicing enhancer (ISE). Conversely, the binding of hnRNPs to exonic splicing silencer (ESS) and intronic splicing silencer (ISS) generally represses exon inclusion. Therefore, splicing factors could compete for the binding in the context of the *cis*-regulatory RNA elements and/or the recruitment of spliceosome component.^22^ In the present study, a simplified spliceosome assembly pathway and the core splicing factors required for exon/intron definition were summarized (Fig. 1).

### Cis-acting elements and other RBPs

These *cis*-acting elements including the aforementioned 5′ SS, 3′ SS, BPS and splice enhancer (ESE, ISE) or silencer elements (ESS, ISS) in precursor mRNA are important for both constitutive and regulated splicing.^23^ The occurrence of alternative splicing is usually accompanied by changes in the ability of sequence-specific RBPs to bind to *cis*-acting sequences in their target pre-mRNA.^24^ Of note, both SR proteins and hnRNPs have been recently reported to be able to either promote or repress splicing when binding to different positions of pre-mRNAs.^21,23,25^ In addition, mutations in these *cis*-acting splice enhancer or silencer elements could largely affect alternative splicing.^26^

In addition to the above core components (snRNPs, SR proteins and hnRNPs), there are hundreds of RBPs genome-widely identified to bind to mRNA, including >300 previously not regarded as RBPs.^27,28^ This information will complicate the understanding of alternative splicing regulation based on competition or combination between different splicing factors and other RBPs [e.g., epithelial splicing regulatory protein 1 (ESRP1), RNA-binding motif proteins 4, 5, 6, 10]. The regulatory complexity of these groups of splicing factors could diversify the alternatively spliced RNA products.

### Main alternative splicing patterns

**Fig. 2:**
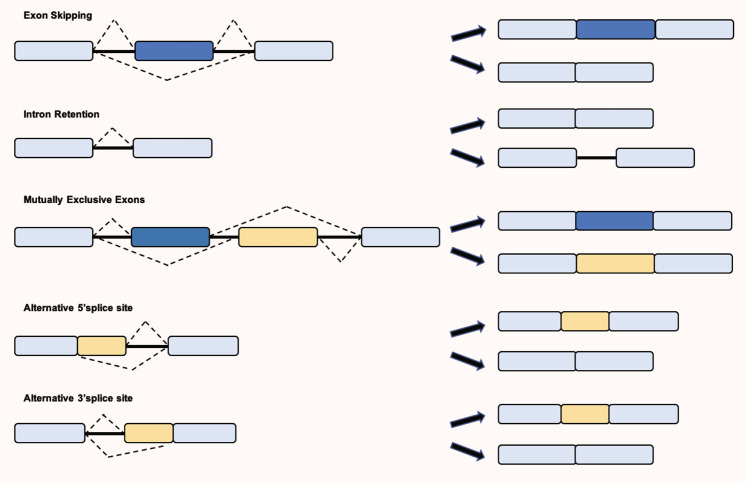
The main alternative splicing patterns are divided into five types: exon skipping (also called cassette exon); intron retention; mutually exclusive exons (only some exons appear in mature mRNA); A5SS (the change of the splicing site causes the position of the 3′ end of the exon to change); A3SS (the change of the splicing site causes the position of the 5′ end of the exon to change). SS, splice site; A5SS, alternative SS

In addition to removing intronic sequences, alternative splicing is also influenced by the RNA structure^29^ and other key regulatory pathways involved in gene regulation, such as transcription rate and the chromatin epigenetic signature.^30,31^ Alternative splicing events could affect one entire cassette exon or part of it through the usage of alternative splicing sites in a variant exon, and occur in genes bearing multiple transcription start sites or using multiple polyadenylation sites. The main alternative splicing patterns are divided into 5 types, as shown in Fig. 2: Exon skipping, intron retention, mutually exclusive exons, alternative 5′ SS and alternative 3′ SS. Growing evidence has revealed that mis-splicing events can change the function of protein and lead to human diseases. The study of its mechanism will provide important information for the treatment of diseases, including cancer initiation, maintenance and/or progression,^32^ which will be discussed in the next section.

## Functions of alternative splicing in cancer

Several studies have reported that alternative splicing is associated with tumors.^33,34^ During the last decades, recurrent somatic mutations in components of the human splicing machinery have occurred in human solid tumors, including bladder,^35^ brain,^36^ breast,^37^ cervix,^38^ colon,^39^ kidney,^40^ liver,^41^ lung,^42^ oral/HN,^43^ ovary,^44^ prostate,^45^ skin,^46^ stomach^47,48^ and thyroid^49^ tumors, as well as hematological malignancies, including acute myeloid leukemia (AML),^50^ myelodysplastic syndrome (MDS),^51^ chronic myelogenous leukemia,^52^ de novo AML,^53^ myelodysplastic syndrome without ringed sideroblasts (MDS w/o RS),^54^ myeloproliferative neoplasm MPN^55^ and refractory anemia with ringed sideroblasts and refractory cytopenia with multilineage dysplasia and ringed sideroblasts (RARS/RCMD).^56^ In addition to cancer, neurological diseases, such as Alzheimer’s disease (AD),^57^ Parkinson’s disease,^58,59^ Huntington’s disease (HD),^60^ schizophrenia,^61,62^ congenital myasthenic syndrome,^63^ Spinal muscular atrophy,^64,65^ and immunological and infectious diseases, such as celiac disease,^66^ psoriasis,^67^ systemic lupus erythematosus,^68^ asthma,^69^ inflammatory response,^70^ viral infections,^71^ cardiovascular disease^72,73^ and diabetes mellitus^74,75^ also have a connection with mis-splicing events (Table 1). Most of the diseases are due to either genetic mutation falling within the canonical RNA splicing sites, which directly influences mRNA maturation, or alterations in the expression level of spliceosomal/splicing regulatory factors that contribute to the splicing of pre-mRNA.

**Table 1:** Alternative splicing in diseases

Diseases	Subtypes		Splicing factors	Spliced isoforms	Biological function	References
Cancer	Solid tumors	Bladder	PTBP1	PKM/MEIS2	Migration, invasion, and proliferation	^35^
		Brain	hnRNPA1	Delta Max	Metabolism	^36^
		Breast	SRSF1	BIM/BIN1	Apoptosis and proliferation	^37^
		Cervix	SRSF10	MIL1RAP	NF-κB activation	^38^
		Colon	SRSF10	BCLAF1	Increased tumorigenic potential	^39^
		Kidney	SRSF1, SRSF2, hnRNP A1	3′UTR	Apoptosis	^40^
		Liver	HBV	HBSP	Constraining inflammation	^41^
		Lung	SRSF1	PTPMT1	Promote phosphorylation of AMPK	^42^
		Oral /HN	SRSF3	Unknown	Metastasis	^43^
		Ovary	hTra2β1, YB-1, SRp20, and ASF/SF2	CD44	Metastasis	^44^
		Prostate	HNRNPF	AR-V7	Proliferation	^45^
		Skin	SRSF6	+E10-15/ΔE14 *Tnc*	Proliferation	^46^
		Stomach	PTBP3	CAV1 PUF60 / FIR	Metastasis proliferation and invasion	^47,48^
		Thyroid	SRSF1	Unknown	Unknown	^49^
	Hematopoietic	AML	SRSF1	Caspase-8	Unknown	^50^
		AML/MDS	SRSF2	Unknown	Unknown	^51^
		CMML	SRSF2	Unknown	Prognostic impact	^52^
		De novo AML	U2AF1	Unknown	Prognostic impact	^53^
		MDS w/o RS	SF3B1	Unknown	Unknown	^54^
		MPN	SF3B1	Unknown	Unknown	^55^
		RARS/RCMD	SF3B1	Unknown	Unknown	^56^
Neurological diseases^19^	Alzheimer’s disease		Unknown	APOE/APP	Unknown	^57^
	Parkinson’s disease		SRRM2	Unknown	Unknown	^58,59^
	Huntington’s disease		SRSF6	Unknown	Unknown	^60^
	Schizophrenia		Unknown	ANK3/ HMGA1a	Unknown	^61,62^
	Congenital myasthenic syndrome		Unknown	AGRN	Unknown	^63^
	Spinal muscular atrophy		Many (e.g., SRSF1, HNRNPA1)	SMN1/2 (exon 7)	Low levels of the SMN	^64,65^
Immunological and infectious diseases	Celiac disease		Unknown	FoxP3	Unknown	^66^
	Psoriasis		Unknown	IL-20R2	Unknown	^67^
	Systemic lupus erythematosus		Unknown	CTLA-4	Unknown	^68^
	Asthma		Unknown	IL-4	Unknown	^69^
	Inflammatory response		Unknown	MD-2	Unknown	^70^
	Viral infections		Unknown	MRP	Unknown	^71^
Cardiovascular disease			RBM20	Unknown	Unknown	^72,73^
Diabetes mellitus			SRSF1	Unknown	Unknown	^74,75^

*AML* acute myeloid leukemia, *AML/MDS* acute myeloid leukemia myelodysplastic syndrome, *CMML* chronic myelomonocytic leukemia, *HN* head and neck, *MDS w/o RS*, myelodysplastic syndrome without ringed sideroblasts, *RARS/RCMD* refractory anemia with ringed sideroblasts and refractory cytopenia with multilineage dysplasia and ringed sideroblasts, *MPN* myeloproliferative neoplasm

**Fig. 3:**
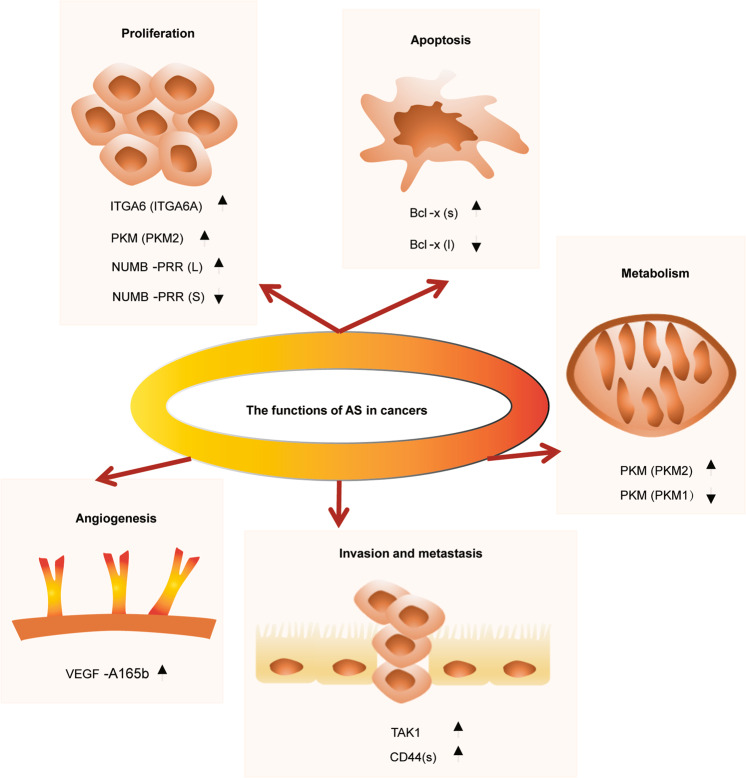
Roles of alternative splicing in tumorigenesis. The diagram illustrates different hallmarks of cancer along with examples of alternative splicing events that contribute to the transformation of normal cells into tumor cells mainly via proliferation and apoptosis, invasion and metastasis, angiogenesis and metabolism. Arrows up and down indicate the alternative isoforms contributing the most and the least to each process. ITGA6 integrin subunit α6, PKM pyruvate kinase, Bcl B-cell lymphoma, VEGF vascular endothelial growth factor, TAK1 TGF-β-activated kinase 1, CD44 cluster of differentiation 44

The transformation of normal cells into cancer cells mainly involves cellular proliferation, escape from cell death, growth inhibition, induction of angiogenesis, invasion and metastasis, energy metabolism and immune escape.^76^ This review focuses on four aspects: Proliferation and apoptosis, invasion and metastasis, and angiogenesis and metabolism under the light of alternative splicing (Fig. 3).

### Balancing between proliferation and apoptosis

Tumor cells have the ability to divide more than they should, and to not die when they should. Therefore, there is a hallmark capability of cells to fine-tune and maintain a balance between proliferation and apoptosis. In general, some tumor suppressor genes, such as p53 and pRb, prevent normal cells from becoming carcinogenic by acting on the cell cycle and promoting genetic changes; it is therefore common for tumor cells to present an aberrant-splicing activity with an increased frequency of splicing isoforms that maintain the abnormal proliferative and apoptotic rhythm.^77^ Alternative splicing participates in the process of proliferation, differentiation and apoptosis via regulating the alternative expression of many oncogenic or tumor suppressor genes,^78^ as well as splicing factors. The integrin subunit α6 (ITGA6) pre-mRNA can be alternatively spliced into two splice isoforms: ITGA6A and ITGA6B. The pro-proliferative ITGA6A variant was enhanced in colon cancer cells, due to a process contributed to by Myc-mediated promoter activation, as well as the splicing factor epithelial splicing regulatory protein 2-mediated alternative splicing, thus promoting cell proliferation.^79^ Similarly, C-Myc can upregulate polypyrimidine tract-binding protein (PTB), hnRNPA1 and hnRNPA2 to change the splicing of pyruvate kinase (PKM) and make it develop into PKM2, which could promote tumor cell proliferation.^80^ Mutations in RNA-binding motif protein 10 (RBM10) had been identified in lung cancer cells, which disrupts the splicing of NUMB and inducing tumor proliferation.^81^ The complex apoptosis mechanisms involve two distinct regulatory pathways: The (extrinsic) death receptor-mediated pathway and the (intrinsic) mitochondrial pathway. Most SR splicing factors, such as RBMP, are associated with pro- and anti-apoptosis effects by influencing caspase 2, B-cell lymphoma (Bcl)-x, myeloid cell leukemia factor 1 (MCL-1), etc.^82^ For example, Bcl-x can obtain two different spliced isoforms, Bcl-xl and Bcl-xs, with Bcl-xl inhibiting apoptosis and Bcl-xs promoting it.^83^ It has been reported that PTB protein 1 (PTBP1)-overexpression can promote the 5′ SS end of the second exon of Bcl-x, which could produce Bcl-xs that promotes apoptosis; conversely, the downregulation of PTBP1 could produce Bcl-xl, which inhibits apoptosis.^84^ Induced MCL-1, as an apoptotic factor of the Bcl-2 family regulators, could cause apoptosis when its expression is decreased, and the alternative splicing of the *Mcl-1* gene can be regulated by SR splicing factor (SRSF)5, as well as SRSF1.^85^ Therefore, the effect of alternative splicing on tumor cell proliferation and apoptosis may provide new therapeutic targets for cancer treatment.

### Invasion and metastasis

Invasion and metastasis are two major obstacles in the treatment of cancer. Epithelial–mesenchymal transition (EMT) is abnormally activated during cancer metastasis and recurrence, which are dependent on interactions between tumor cells and the microenvironment.^86^ It may be important to define whether a tumor is invasive or metastatic, in order to determine its behavior. Transforming growth factor-β (TGF-β), as the main inducer of EMT, induces alternative splicing of TGF-β-activated kinase 1 to reject exon 12 during EMT.^87^ In EMT, the alternative splicing of cluster of differentiation 44 (CD44) has been changed through the regulation of the splicing factor ESRP1, which determines the interaction between CD44 and cell surface receptor tyrosine kinases.^88^ The generated CD44s could promote the occurrence and development of the EMT of breast cancer cells. ESRP1 and hnRNPM could compete for GU-rich binding sites in the pre-mRNA and modulate exon inclusion or skipping, defining either an epithelial or a mesenchymal state to determine a specific cell fate.^89^ It was recently revealed that hnRNPM and ESRP1 are key regulators in the EMT splicing program and are correlated with breast cancer.^90^

### Angiogenesis

Angiogenesis is one of the critical features of tumor progression through the formation of new blood vessels. Many proteins act as vascularization activators, including basic fibroblast growth factor, tumor necrosis factor-α and vascular endothelial growth factor (VEGF). VEGF-A is composed of 8 exons, and exons 6, 7, and 8 alternately select 3′ and 5′ SSs to produce isoforms, which can control angiogenesis. VEGF-A could lead to both angiogenic and anti-angiogenic results, and might thus be utilized for anti-angiogenic therapeutics.^91^ It has been reported that inhibiting serine/arginine protein-specific splicing factor kinase 1 (SRPK1) by downregulating the tumor suppressor factor Wilms’ tumor suppressor 1 and indirectly suppressing SRSF1, could turn the splicing of VEGF into VEGF120 with an anti-angiogenic effect, thus inhibiting the growth of tumor endothelial cells.^92^ The evidence from a murine retinal model indicated that SRPK inhibitor 1 (SRPKIN-1), as an inhibitor of SRPK, can regulate the splicing of VEGF and then transform it into an anti-angiogenic VEGF-A165b isoform.^93^ Truncated glioma-associated oncogene homolog 1, as a splice variant of the *GLI1* gene, could upregulate the expression of VEGF-A, thus enhancing the angiogenesis of glioblastomas.^94^ The alternative splicing of other factors in angiogenesis, in order to provide all the necessary nutrients for tumor development, need to be further demonstrated.

### Alternative splicing in tumor metabolism

During tumor progression, cancer cells may experience nutrient deprivation and hypoxia, due to overgrowth. As compared with normal cells, tumor cells preferentially metabolize glucose through the aerobic glycolysis pathway, for which some genes encoding enzymes are susceptible to alternative splicing. The last step in glycolysis is the conversion of phosphoenolpyruvate to pyruvate, is catalyzed by PKM, which are encoded by two genes: PKLR and PKM, each producing two different variants.^95,96^ PKLR is mainly expressed in liver and hematopoietic cells, but the majority of tissues express PKM, coding PKM1 and PKM2 variants.^97^ Through alternative splicing of two mutually exclusive exons, exons 9 and 10, PKM1 is constitutively expressed in normal cells while PKM2 is expressed in tumor cells in a cell-signal-dependent manner.^98^ A study has shown that the alternative splicing of PKM is regulated by splicing factors: hnRNPA1/hnRNPA2, PTB/nPTB, and SRSF3,^99^ suggesting the role of splicing factors involved in alternative splicing for tumor metabolism. The upregulation of PKM2 is common in several types of cancer and contributes to a switch to glycolytic metabolism, reflecting the modulation of PKM1 and PKM2 ratio by alternative splicing to determine the choice between aerobic glycolysis and mitochondrial oxidative phosphorylation in response to metabolic stress.

Another aspect of tumor metabolism affected by alternative splicing events is the cellular response to hypoxia, which is a common characteristic of the inner bulk of many solid tumors. A recent RNA-seq study showed that breast cancer cells underwent extensive alternative splicing changes when cultured in hypoxic conditions, such as predominant intron retention events (for LDHA, TNFSF13 and ARHGAP4), as well as exon skipping (for MARCH7, PCBP2, and LRCH3).^100^

## Alterations of splicing regulatory factors and related signal transduction pathways in tumor progression

During alternative splicing under the regulation of certain splicing factors, specific splicing isoforms are produced.^101^ In tumors, especially in hematological malignancies, abnormal changes in splicing factors often occur.^102^ Hematological tumors are malignant clonal diseases of hematopoietic cells, with genetic mutations in ~85% of patients.^103^ This section contains a review of the detailed changes in core splicing factors SRSF2, SF3B1, U2AF1 and ZRSR2,^104,105^ since these are the most frequently mutated, as well as the contribution of other RBPs under the settings of related gene-regulatory network.

### Aberrant expression of core splicing factors

SRSF2 (also as known as SC35) is rich in serine and arginine. As early as in 1990, SRSF2 was first discovered in HeLa cells and was identified as a non-snRNP protein-splicing component.^106^ The SRSF2 mutations often occur in the linking sequence of the RNA-recognition motifs (RRM) and RS regions, the mutations of which affect exons by recruiting U2AF to the upstream 3′ SS and U1 snRNP to the downstream 5′ SS.^107,108^ Studies have shown that mutations in SRSF2 have been found in hematological malignancies, with the highest frequency occurring in chronic myelomonocytic leukemia (CMML; 28–47%), and the lowest in MDS.^109^

Repeated mutations of the splicing factor SRSF2, such as P95 hotspot, often occur in hematological malignancies.^110^ Liang et al.^111^ confirmed the differential splicing of several hnRNP proteins by constructing an SRSF2^P95H^ mutant cell line. The results showed that mutations in splicing factors could induce hematopoietic differentiation by affecting hnRNPA2B1 protein function, thereby promoting cancer development. Zhang et al.^112^ introduced common mutations in MDS into SRSF2. The results showed that the mutation of the splicing factor SRSF2 erroneously regulated the splicing of the pre-mRNA and produced abnormal proteins that could drive cancer.

SF3B1 (SF3B155) is a key component of U2 snRNP in the spliceosome. It binds to intron branch SSs and affects the 3′ SS. The most common mutation hotspots are K700, E622, R625, H662, and K666. SF3B1 has significant mutations not only in hematological malignancies, but also in solid tumors, which occur in 48–79% of RARS and RCMD-RS 6–26% of chronic lymphocytic leukemia (CLL).^109^

Mutations of SF3B1 were first found in patients with MDS.^113^ It was also found that mutations in SF3B1 were closely associated with the presence of ring sideroblasts in MDS, suggesting that mutations in SF3B1 are associated with refractory anemia with RARS.^56^ Wang et al.^114^ found that mutations in SF3B1 caused dysfunction in cells, including DNA damage, affecting Notch signaling, etc., thus indicating that it is associated with poor prognosis of CLL.

U2AF1 is also known as U2AF35. It can directly bind to the 3′ SS,^115^ with S34 and Q157 as the mutation hotspots. U2AF1 mutations mainly occur in hematological tumors, especially MDS (5–12%), CMML (8–17%)^109^ and lung cancer (3%).^116^ The transgenic mouse model showed that the mutations in U2AF1 altered the function of mouse hematopoietic cells, and simultaneously changed the splicing of precursor mRNA and the expression of downstream gene subtypes, U2AF1 (S34F) compared with U2AF1 (WT) transgenic mice, resulting in abnormal hematopoietic function in patients with MDS.^117^ The mutation of the S34 mutation hotspot in U2AF1 causes abnormal alternative splicing, affecting gene expression through canonical or non-canonical roles of translation regulation.^118,119^

ZRSR2 is also a member of the arginine and serine rich family and affects the 3′ SS through U12. ZRSR2 mutations are evenly distributed throughout the gene sequence, so there are no mutation hotspots. ZRSR2 mutations also mainly occur in hematological tumors. ZRSR2 have smaller mutations in MDS (1–11%) and CMML (0.8–8%), as compared to SRSF2, SF3B1 and U2AF1.^109^ By knocking down ZRSR2 in leukemia cells, which showed a slow growth trend, as compared with the control group, confirming the special role of ZRSR2 in RNA splicing and the U12 intron splicing disorder MDS.^120^ Of note, the three components of the major spliceosome (SF3B1, SRSF2, and U2AF1) generally occur in a mutually exclusive manner as heterozygous change-of-function mutations; ZRSR2 does not follow this pattern, suggesting an unknown association between the four spliceosome gene mutations and their potential roles in oncogenesis, which are unique to the different subtypes of hematological malignancies.^121^

According to reports, alterations in other splicing factors, such as PRPF8, SF3A1, LUCL7L2, SF1, U2AF2, PRPF8, PRPF40B, SRSF6, SRSF1, SRSF7, TRA2β, SRRM2, DDX1, DDX23, and CELF4^108,122–125^ have very low frequencies among hematological tumors.

### Contribution of other RBPs to dysregulated alternative splicing

The expression of many RBPs can be autoregulated by the splicing of their own pre-mRNA, with the enhanced inclusion of premature termination codon-containing cassette exons, termed “poison exons”. These transcripts could be degraded by a nonsense-mediated decay (NMD) method, forming a negative feedback loop when SR-protein levels are upregulated.^126,127^ The coupling of splicing-factor-mediated alternative splicing with NMD in tumors needs further investigation. Furthermore, many other RBPs have been reported to be involved in the dysregulated alternative splicing of cancer cells.^27,28^ Herein hnRNPK and RNA-binding motif proteins were used as examples.

hnRNPK is an RBP containing three K homology domains (KH1, KH2 and KH3) and is closely associated with tumors.^128^ hnRNPK could play either a tumor suppressive or an oncogenic role in different types of cancer. hnRNPK has been shown to contribute to leukemogenesis in AML patients with deletions on the long arm of chromosome 9, which is correlated with a reduced expression of hnRNPK.^129^ However, another study found that hnRNPK is overexpressed in diffuse large B-cell lymphoma patients without Myc genomic alterations, representing a mechanism of C-Myc activation without Myc lesions, due to hnRNPK overexpression.^130^ In addition, the overexpression of hnRNPK can promote lung metastasis,^131^ and the increased hnRNPK expression is observed in multiple types of cancer, including breast, prostate, colorectal, gastric and pancreatic cancers.^132^ Therefore, considering the inhibition of tumor cell growth by hnRNPK as a haploinsufficient tumor repressor in myeloid malignancies,^133^ it is tempting to speculate that the dichotomous roles of hnRNPK in tumorigenesis may reflect the diverse hnRNPK functions in different cellular contexts. However, the exact molecular mechanisms of hnRNPK in tumorigenesis remain unclear and require further study.

RNA-binding motif proteins 4, 5, 6, and 10 are RBPs, which can regulate alternative splicing. In breast cancer cells, elevated SRPK1 induces cytoplasmic accumulation of phosphorylated RBM4, and eliminates its pro-apoptotic effect by inducing RBM4-regulated splicing transcripts of *IR-B* and *MCL-1S*.^134^ Downregulated RBM5 inhibits bladder cancer cell apoptosis.^135^ RBM6 could reduce the expression of EGFR, extracellular signal regulated kinase (ERK) and phosphorylated (p)-ERK in vitro and in vivo, thus repressing the growth and progression of laryngocarcinoma.^136^ Studies have shown that RBM10 inhibits the proliferation of endometrial cancer.^137,138^ Conversely, in lung adenocarcinoma, RBM10-overexpression reduced p53 expression in A549 cells and inhibited apoptosis;^139^ therefore, RBM10 could also promote lung adenocarcinoma cell proliferation through the RAP1/AKT/CREB signaling pathway.^140^ Many other RBPs, as splicing factors, need to be further characterized, especially in the setting of the complicated gene-regulatory network of alternative splicing.

### Signal transduction driving alternative splicing during tumorigenesis

**Fig. 4:**
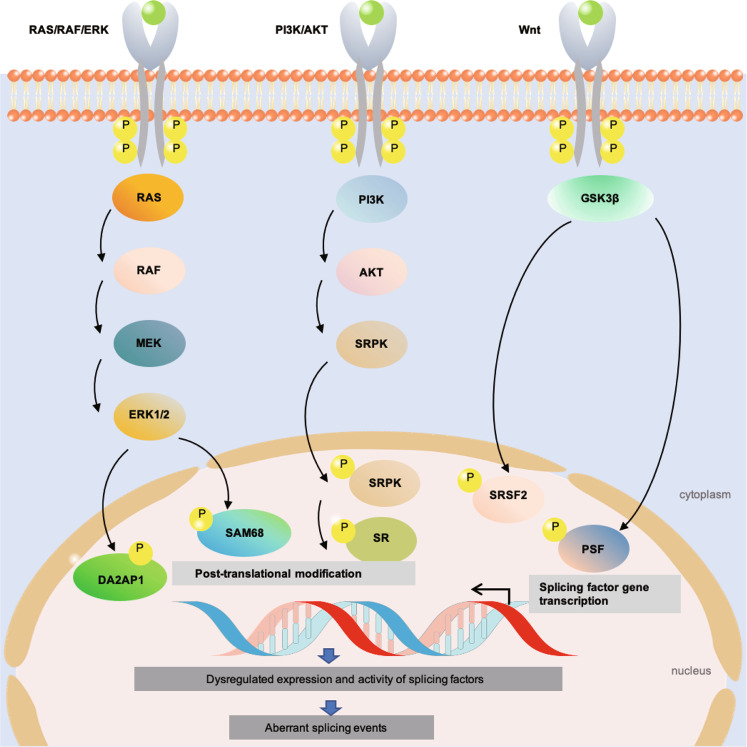
Oncogenic signal transduction driving alternative splicing in cancer. The RAS/RAF/ERK, PI3K/AKT and Wnt signaling pathways play an important role in regulating splicing factor via transcriptional regulation and/or post-translational modification, which lead to aberrant-splicing events that promote oncogenesis. P phosphorylation, Wnt Wingless, ERK extracellular signal regulated kinase, PI3K phosphatidylinositol 3-kinase

Although alterations in some splicing factors (snRNPs, SR proteins and hnRNAPs) have been described, a major driver of dysregulated splicing is oncogenic signal transduction pathways. Signaling could affect the expression of splicing factors at either the transcriptional or the post-translational modification levels, both of which modulate the subcellular localization to the nucleus or cytoplasm in a given cell. For example, in the process of spliceosome assembly and catalysis, the activity of SR proteins is usually regulated by phosphorylation.^23^ SRPKs in the cytoplasm and CDC-like kinases in the nucleus are two families of kinases involved in the regulation of SR-protein phosphorylation.^141^ These biochemical events are usually chained to transmit signals from receptors with upstream ligand stimulating downstream effectors inside the cell. Aberrant transduction of such signaling pathways is common during tumorigenesis and provides promising targets for drug development to control the growth and survival of malignant cells. These cancer cell intrinsic alterations were found to cause dysregulated alternative splicing, as will be briefly discussed in this section (Fig. 4):

The RAS/RAF/ERK pathway, which is characterized by the activation of the small GTPase RAS with a cascade stimulation of three mitogen-activated protein kinases (MAPK; namely RAF, MEK and ERK), is a hallmark event in many epithelia cell-derived tumors. Transcriptomic RNA-seq data from colon adenocarcinoma or lung squamous cell carcinoma showed that the oncogenic KRAS was correlated with an ETS transcription factor-mediated aberrant-splicing regulatory network. The network involved the induction of the expression of the alternative splicing factor PTBP1, which was associated with a shift in the alternative splicing of transcripts encoding the small GTPase RAC1, adaptor protein NUMB and PKM.^142^ The MEK/ERK signaling pathway was also reported to mediate the phosphorylation of splicing activator DAZAP1, which is required for its cytoplasm-to-nucleus translocation, as well as splicing regulatory activity.^143^ In addition, the activation of the MAPK/ERK downstream of RAS could cause the phosphorylation of splicing factors, such as signal transduction and activation of RNA metabolism 68 (SAM68). Phospho-SAM68 could then bind to and promote an intron retention event in the 3’UTR transcript of the SRSF1, thus leading to the subsequent switch in splicing profile, such as the RON gene transcripts.^144,145^ Of note, it was recently reported that the increased hnRNPA2 expression in hepatocellular carcinoma could induce an alternative splicing switch that decreases the dominant-negative isoform of A-RAF, leading to the activation of the RAF/MEK/ERK pathway and cellular transformation.^146^ This example highlights that the manipulation of certain alternative splicing variants can also shape cell signaling or even cell fates, which could be exploited to investigate the feedback regulatory mechanisms or therapeutic uses (see section 6).

The phosphatidylinositol 3-kinase (PI3K)/AKT pathway is another key pathway involved in regulating cell survival and apoptosis in a variety of tumors. For example, activated AKT has been shown to phosphorylate SRSF1 in lung cancer cells, thereby generating an anti-apoptotic caspase-9b isoform through the exclusion of an exon 3,4,5,6 cassette.^147^ EGF signaling was reported to regulate splicing through the phosphorylation of AKT/SRPK/SR protein^148^ and/or SPSB1-hnRNPA1 ubiquitination.^149^ The PI3K/AKT pathway is also known to activate the mammalian target of rapamycin complex 1 (mTORC1), a key regulator of cell metabolism and growth. The signaling of the mTORC1-S6K1 axis promotes the phosphorylation and cytoplasm-to-nucleus translocation of kinase SRPK2, which further activates SR proteins to participate in the splicing of lipogenesis-related transcripts to fuel tumor cell metabolism.^150^

Wingless (Wnt) signaling is well known for regulating development and stemness, and for its close association with many cancer types, especially colorectal cancer (CRC).^151^ Glycogen synthase 3β is part of the canonical Wnt pathway and has been reported to direct the phosphorylation of splicing factors, such as SRSF2^152^ or PTB-associated splicing factor (PSF).^153^ Activated Wnt/β-catenin signaling could directly enhance the transcript level of SRSF3.^154^ SRSF3 has also been reported to negatively affect one alternative exon inclusion variant of RAS-related C3 botulinum toxin substrate 1b.^155^

In addition to the three signaling pathways described above, there are many other tumor microenvironment-derived soluble factors, metabolic stress conditions and extracellular matrix-related signals, which also have a crosstalk with alternative splicing and are not discussed here.^155^ Therefore, more detailed studies are required to investigate the intricate interplay between oncogenic signaling pathways and dysregulated splicing in cancer.

## Connection between mis-regulated splicing events of non-coding RNAs and cancer

During the past decades, thousands of non-coding RNAs (ncRNAs), including long ncRNAs (lncRNAs) and circular RNAs (circRNAs), have been characterized and found to play important biological roles in many cellular aspects, including cancers.^156,157^ Some mis-spliced RNA transcripts may also contribute to tumorigenesis.

### lncRNAs and circRNAs

lncRNAs belong to an RNA subgroup and are usually longer than 200 nucleotides, have an important regulatory effect on cellular metabolism and are involved in the regulation of alternative splicing.^158,159^ At present, many studies have shown that the occurrence of diseases is associated with the mis-splicing of lncRNAs (Table 2). Serine/threonine-protein phosphatase 1 regulatory subunit 10 (lncRNA-PNUTS) is generated with the involvement of hnRNPE1, a non-coding isoform of PNUTS, and could functionally serve as a competitive sponge for miR-205 in breast tumor implantation, growth and metastasis.^160^ lncRNA HOXB-AS3 could interact with the ErbB3-binding protein 1 (EBP1) and regulate ribosomal RNA transcription and de novo protein synthesis in NPM1-mutated AML.^161^ The knockdown of metastasis-associated lung adenocarcinoma transcript 1 (MALAT1) in ovarian cancer led to a reduced expression of RBP fox-1 homolog 2 (RBFOX2), thus inhibiting tumor cell proliferation.^162^ MALAT1 could also release SFPQ (also known as PSF (PTB-associated splicing factor)) from SFPQ/PTBP2 complex by binding to SFPQ to promote tumor growth and migration in CRC.^163^ In CRC, LINC01133 interacts with SRSF6 to inhibit EMT and metastasis.^164^ HOX transcript antisense RNA (HOTAIR) is an indicator of cell cycle dysregulation in lung cancer.^165^ An increasing number of mis-spliced lncRNAs are being reported in other diseases, such as adipogenesis,^166^ retinal diseases,^167^ Prader-Willi syndrome,^168^ and AD.^169^

**Table 2:** Diseases related to mis-spliced lncRNAs

Disease		lncRNA	Mechanism	References
Cancer	Breast	PNUTS	Related to hnRNP E1, regulate the metastatic potential of tumor cells	^160^
	AML	HOXB-AS3	Regulates ribosomal RNA transcription in NPM1-mutated AML	^161^
	Ovarian cancer	MALAT1	Knockdown MALAT1 in ovarian cancer leads to reduced expression of RNA-binding protein fox-1 homolog 2 (RBFOX2) to inhibit tumor cell proliferation	^162^
	CRC (colorectal cancer)	MALAT1	Releasing SFPQ from SFPQ / PTBP2 complex by binding to SFPQ to promote tumor growth and migration	^163^
		LINC01133	Interacting with SRSF6 to inhibit the EMT and metastasis	^164^
	Lung	HOTAIR	Indicator of cell cycle dysregulation	^165^
Adipogenesis		NEAT1	Associates with SRp40 to regulate PPARγ2 Splicing	^166^
Retinal cell		RNCR2	Regulating retinal cell differentiation.	^167^
Prader-Willi syndrome		SPA	Patterns of RBPs binding could be altered and alternative splicing.	^168^
Alzheimer		17A	Embedded in G-protein-coupled receptor 51 gene (GPR51), result the abnormal function of GABA B and alternative splicing	^169^

circRNAs are widely expressed endogenous RNAs via a back-splicing process, which is the covalent joining of a downstream splice donor site with an upstream splice acceptor site.^156^ Since its biogenesis is performed by the canonical spliceosome machinery and regulated by the same *cis*-regulatory elements and *trans*-acting factors as that control linear mRNA splicing, circRNAs can be regarded as an additional form of alternative splicing,^170^ and in fact certain circRNAs are expressed in the context of cancer-specific alternative spicing.^171^

circRNAs may regulate multiple biological processes, and their aberrant expression has been reported in several types of cancer, even if the majority of circRNAs still lack functional annotations.^172^ Recent reports have identified their potential in gene expression regulation at the transcription and post-transcription levels through their interaction with the elongating RNA polymerase II complex^173^ or the U1 snRNP.^174^ Furthermore, circRNAs biogenesis may compete with pre-mRNA maturation, thus decreasing the levels of linear mRNAs that harbor circularized exons.^175^ In addition, circRNAs can function as sponges for microRNAs (miRNAs)^176^ to sequester normal functional miRNA. It may also act as a decoy for RBPs to regulate intracellular mRNA fate,^177^ or even produce abnormal small peptides if they contain internal ribosome entry site elements and AUG initiator sites in the open-reading frame, thus expanding the regulatory repertoire of circRNA.^178,179^ It is therefore tempting to speculate that circRNAs, as well as other RNA spliced variants, could be used as potential biomarkers for cancers, such as leukemia and lung cancer.^180,181^

### Mis-regulated splicing events acting as oncogenic drivers and passenger factors in tumorigenesis

The above description indicates that mis-splicing events could be generally regarded as oncogenic drivers, which is indeed supported by recent multi-omic data from large-scale samples.^182–184^ However, aberrant spliced transcripts may also act as bystander or passenger factors in tumorigenesis. A recent study on multiple myeloma, using unbiased phosphoproteomics and transcriptomic/exomic sequencing following treatment with the proteasome inhibitor carfilzomib, found that the splicing-related proteins underwent the most prominent phosphorylation changes, and that the spliceosome might be a specific vulnerability in this hematological malignancy.^185^ Kahles et al.^186^ have provided a landscape of the splicing differences between the most common tumors and the healthy counterpart samples from 8,705 patients, suggesting that the increased splicing diversity in tumors might be an effect rather than a cause of oncogenesis. They also highlighted that certain cancer-specific alternative splice isoforms could produce neo-antigens, which can be used as a novel strategy for cancer immunotherapy (see next section).

## Modulation of splicing as cancer therapeutics

**Fig. 5:**
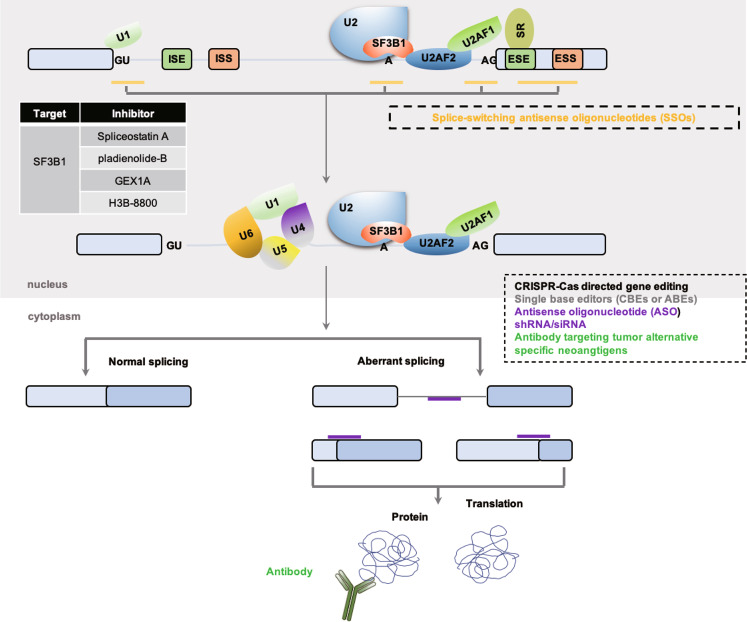
Modulation of splicing as potential cancer therapeutics. Several representative strategies are depicted in the simplified splicing regulation diagram, including small molecules targeting the core spliceosome SF3b-complex compound, SSOs, ASO, short hairpin RNA interference/small interference RNA, clustered regularly interspaced short palindromic repeats (CRISPR)-associated (Cas) system, single-BEs (CBEs or ABEs) as well as antibodies against tumor-specific neo-antigen due to alternative splicing. SR serine/arginine-rich, ESE exonic splicing enhancer, ESS exonic splicing silencer, ISE intronic splicing enhancer, ISS intronic splicing silencer, SSOs splice-switching antisense oligonucleotides, ASO antisense oligonucleotide, BEs base editors, CBEs cytosine-BEs, ABEs adenine-BEs, CRISPR-Cas clustered regularly interspaced short palindromic repeats-Cas

As mentioned earlier, there is an important link between alternative splicing and cancer.^187^ Abnormal alternative splicing, which may produce abnormal proteins, could be explored as a marker for cancer diagnosis^188^ and a new target for cancer treatment.^189^ At present, small molecules and splice-switching antisense oligonucleotides (SSOs) are two major validated methods that target alternative splicing for the treatment of cancer (Fig. 5).

### Small molecules modulating the levels of splicing regulators

Since some specific splice isoforms are used as potential tumor markers and therapeutic targets, it is reasonable to design inhibitors targeting splicing factors.^190^ In fact, SF3B1, having the highest mutation degree in various hematological malignancies, is often used as a target for cancer treatment. Some small molecules, such as spliceostatin A, pladienolide-B, GEX1A and E1707, can inhibit the splicing of SF3B1, and have a selective effect on tumor cells.^191^ For example, Amiloride is a small molecule first discovered in the 1960’s, which has been used clinically to treat edema and hypertension.^192^ It has been validated that amiloride can change the alternative splicing of Bcl-x, HIPK3 and RON/MISTR1 by affecting hypo-phosphorylation of splicing factor SF2/ASF and decreasing the expression of SRP20 and other SR proteins.^193^ H3B-8800 is an orally available small-molecule splicing modulator derived from pladienolide-B with a structural similarity to E7107 but with less potency, which interacts directly with the splicing factor SF3b complex to kill spliceosome-mutated epithelium and blood tumor cells by causing enhanced retention of GC-rich introns.^194^ H3B-8800 has recently been used in a phase 1 clinical trial (NCT02841540) targeting patients with relapsed/refractory myeloid neoplasms (MDS, CMML and AML) that carry splicing factor mutations (https://clinicaltrials.gov/ct2/show/NCT02841540).

Although the effects of small-molecule drugs are significant, they have an obvious disadvantage: They easily miss targets. Therefore, further clinical research on these small-molecule drugs is required to ensure that their mechanism of action is fully understood.^121^

### SSOs

SSOs can affect the function of splicing factors through splicing modification, which has become an effective treatment method.^181,195^ The prototype for SSO technology is the antisense oligonucleotides (ASOs), which have been approved for the treatment of spinal muscular atrophy (SMA)^196^ and Duchenne muscular dystrophy.^197^ SMA is a congenital neuromuscular disease characterized by the loss of motor neurons, which leads to progressive muscle weakness and is difficult to cure.^198^ Many efficient ASOs are under development or have already been tested in clinical trials for the treatment of myotonic dystrophy (NCT023412011), HD (NCT02519036), amyotrophic lateral sclerosis (ALS; NCT02623699) and AD (NCT03186989).^199^

This base-pairing of oligonucleotides to target RNA can induce degradation or interfere with the splicing of pre-mRNA. In order to improve the stability of synthetic oligonucleotides, replacing the ribose ring of the oligonucleotide subunits with a morpholine ring, termed morpholino,^200^ seems especially suitable for targeting splicing, as termed morpholino are refractory to RNase H activity and thus not directly degrade the pre-mRNA. Studies have shown that Bcl-x SSOs could be combined with the downstream 5′ SS of the exon 2 in pre m-RNA of *Bcl-x* and modify Bcl-x pre-mRNA splicing. The pro-apoptotic effect on tumor cell lines demonstrates the anti-tumor activity of Bcl-x pre-mRNA spliced SSO.^201,202^ The decoy RNA oligonucleotides were designed and confirmed to inhibit the splicing and biological activity of RBFOX1/2, SRSF1 and PTBP1.^203^ Therefore, SSOs will be an effective way to treat tumors caused by the vital mis-spliced events during disease initiation and/or progression. Ongoing efforts to discover pathogenic isoform alterations are being made to exploit the full potential of this therapeutic approach.

### Generation of alternative tumor-specific antigens for cancer immunotherapy

The advent of immunotherapy has revolutionized the treatment of many solid tumors, as well as blood cancers, and the research of suitable antigens specific to tumors for cancer vaccines and T cell therapies has accelerated but remains a challenge within the past decade. Based on certain different patterns of alternative splicing of mRNA between tumors and normal tissues, the development of neo-antigens that are produced by aberrant spliced mRNA and are not recognized by the immune system, including splice variants, gene fusions and other processes,^204^ would be a novel strategy. Over the past decades, there have been a few experimentally validated splicing-derived peptides with neo-epitopes that are recognized by T cells with evidence of immunogenicity. In a study on chronic myeloid leukemia, peptides derived from alternatively spliced out-of-frame BCR/ABL fusing transcripts were able to stimulate a peptide-specific cytotoxic T lymphocyte response, evidenced by the detection of out-of-frame peptide-specific IFNγ + CD8 + T cells in patients and the killing of peptide-pulsed target cells in vitro by these cytotoxic T lymphocytes.^205^ Another recent study on B-cell lineage marker CD20 showed that its alternative splicing isoform with a 168-nucleotide spliced out in exons 3–7 was only present in several patient-derived B lymphoma cell lines but not normal cells, and could generate a CD20-derived peptide with HLA-DR1 binding epitopes and vaccination, thus eliciting epitope-specific CD4+ and CD8+ responses in transgenic mice.^206^ Although there are many computational tools available and high-throughput studies to predict, screen and validate the presentation and immunogenicity of generated peptides, the field of investigating tumor-specific-splicing events as a promising anticancer treatment strategy remains in its nascence and presents many challenges, such as specific and cross-reactive immunogenicity, as well as tumor clonal heterogeneity (see recent more comprehensive reviews^207,208^).

### Other RNA-based therapeutic approaches

Targeting mis-spliced RNA transcripts during tumorigenesis remains relatively nascent. Novel technologies such as short hairpin RNA interference, small interference RNA,^209^ clustered regularly interspaced short palindromic repeats (CRISPR)-Cas directed gene editing,^210^ and even single-base editors (BEs) cytosine-BEs (CBEs) or adenine-Bes (ABEs) represent powerful and exciting strategies.^211,212^ Recently, the CRISPR-Cas13a enzyme could target and edit RNA transcripts,^213,214^ such as endogenous post-transcriptional RNA editing and alternative polyadenylation, representing another approach to targeting RNA molecules from variant or aberrant-splicing processes with a potentially great clinical utility in the future, once its feasibility and safety have been established in humans.

## Conclusion and future perspectives

In conclusion, alternative splicing can produce multiple isoforms with diverse functions for the same gene loci. The dysregulation of alternative splicing is closely associated with tumor progression, which is becoming a hot topic in the field of cancer research, providing new ideas for attractive methods of treating cancer. In-depth studies of the regulatory mechanism of alternative splicing have extended our understanding of tumorigenesis. In the light of high-throughput data of the genome, transcriptome, proteome and epigenome from large-scale samples and functional characterization, the aberrant-splicing events could be involved in tumorigenesis as oncogenic drivers and/or passengers. Targeted splicing has also been explored as a new treatment option for cancer and other diseases, including novel RNA-based CRISPR-Cas13a editing technology, SSOs, as well as small molecules that modulate splicing. By knocking down PTBP1 using ASOs or CRISPR-CasRx technologies, two recent papers successfully converted glial cells to neurons in mouse models of Parkinson’s disease.^215,216^ However, more recent studies have shown that one of the major challenges of targeting the splicing events and/or aberrant RNA species at the post-transcriptional level are the specificity and delivery efficiency.

Therefore, exploring the mutation site and studying the abnormality caused by mis-splicing in cancer is of great importance, as it may provide novel ideas for the follow-up research of splicing-targeting drugs and improve the promising RNA-based anti-tumor therapy.

## Supplementary information

certificate
