# Supplementary material for: Alternative splicing and cancer: a systematic review
Source: Signal Transduct Target Ther. 2021 Feb 24;6:78. doi: 10.1038/s41392-021-00486-7 (PMC7902610; doi:10.1038/s41392-021-00486-7)
Supplement: Supplementary file 1 — certificate [file 41392_2021_486_MOESM1_ESM.pdf]

SPANDIDOS PUBLICATIONS

EDITORIAL CERTIFICATE

This document certifies that the manuscript listed below was edited for English language, grammar, punctuation, spelling and overall style by one or more editors at Spandidos Publications English Language Editing Service

MANUSCRIPT TITLE:

Alternative splicing and cancer: A systematic review

AUTHORS:

Yuanjiao Zhang et al

SECTIONS EDITED:

Whole manuscript (excluding Author List, Affiliations, Correspondence information, Key words, Declarations, References, Table contents, Table legends)

DATE ISSUED:

03/08/2020

CERTIFICATE VERIFICATION NUMBER:

ESUB-7026

CERTIFICATE OF LANGUAGE EDITING
SPANDIDOS PUBLICATIONS

Neither the research content nor the authors' intentions were altered in any way during the editing process. Documents receiving this certification should be English-ready for publication; however, the authors have the ability to accept or reject our suggestions and changes. This certificate may be verified at https://www.spandidos-publications.com/langeediting/publiclanguageediting/certificatedetails?submissionId=7026&uniqueNumber=1870b5b9a0d72f2c5daf36e8a90343cf. If you have any questions or concerns regarding the edited document, please contact Spandidos English Language Editing Service at languageediting@spandidos-publications.com, quoting the certificate verification number. Spandidos English Language Editing provides a high quality range of services for researchers to prepare manuscripts ready for journal submission. For more details regarding our company and current services, please visit: http://www.spandidos-publications.com/.
